# Validation of the Madrid Genotype Score to Predict Pathogenic Variants in U.S. Dilated Cardiomyopathy Patients

**DOI:** 10.1016/j.jacadv.2026.102648

**Published:** 2026-03-13

**Authors:** Rebecca Resnick, Eric Y. Kao, Charles Maynard, Shiva Tabaghi, Andrew B. Stergachis, Babak Nazer, Kai-Chun (Daniel) Yang

**Affiliations:** a Division of Cardiology, University of Washington, Seattle, Washington, USA; b Division of Medical Genetics, University of Washington, Seattle, Washington, USA; c Department of Health Systems and Population Health, University of Washington, Seattle, Washington, USA; d Department of Genome Sciences, University of Washington, Seattle, Washington, USA; e Brotman Baty Institute for Precision Medicine, University of Washington, Seattle, Washington, USA; f Institute for Stem Cell and Regenerative Medicine, University of Washington, Seattle, Washington, USA; g Center for Cardiovascular Biology, University of Washington, Seattle, Washington, USA; h Cardiology/Hospital Specialty Medicine, VA Puget Sound HCS, Seattle, Washington, USA

**Keywords:** dilated cardiomyopathy, genetic testing, pathogenic variants

**What is the clinical question being asked?**

Does the Madrid Genotype Score apply to the U.S. population?

**What is the main finding?**

The Madrid Genotype Score achieved modest diagnostic genetic testing yields in a U.S. dilated cardiomyopathy cohort that were comparable to those reported in European cohorts.

Contemporary guidelines provide a class IIa recommendation to consider genetic testing for patients with dilated cardiomyopathy (DCM); however, there is limited guidance regarding which patients should be tested.^1^ Identification of pathogenic variants can stratify risk for sudden cardiac death, inform family planning, and enable cascade screening of at-risk relatives. Although approximately 25% of patients with DCM have an underlying identifiable causative genetic variant, fewer than 1% of U.S. patients with DCM undergo genetic testing.^2^, ^3^, ^4^

The Madrid Genotype Score (MGS) is a simple 5-point score of clinical characteristics that was developed to help predict the likelihood of finding causative variants in patients with DCM and thereby increase the yield for genetic testing.^3^ However, this score has not been validated in cohorts outside of Europe, and no threshold score has been proposed to guide referral for genetic testing. If further validated, the MGS could be part of the routine workup for patients with DCM in the United States. To assess the applicability of the MGS to a U.S. population and explore opportunities for improvement, we used the Brotman Baty Institute Clinical Variant Database (BBI-CVD), which contains genetic testing results for patients seen by University of Washington clinical geneticists.

Patients in the BBI-CVD with DCM diagnosed in adulthood who underwent cardiomyopathy gene panel testing were included (n = 143). Panels included >50 genes and were from Invitae (83%), GeneDx (8.5%), or University of Washington/Northwest Clinical Genomics Laboratory (8.5%). Of these, 49 patients had diagnostic testing (pathogenic/likely pathogenic variants), 69 had variants of uncertain significance, and 25 had nondiagnostic testing (no variant, benign/likely benign variant, or a pathogenic variant that could not explain their clinical presentation). Variants of uncertain significance were combined with nondiagnostic testing for analysis as they are not clinically actionable. Chart review was performed to identify MGS variables (family history of DCM, skeletal myopathy, no hypertension at diagnosis, no left bundle branch block, and low voltage on electrocardiogram) along with other clinical variables that we hypothesized may improve the MGS: complete heart block, sustained ventricular tachycardia, expanded family history of DCM (including family history of any nonischemic cardiomyopathy and not specifically DCM), and pregnancy-associated cardiomyopathy within 1 year of pregnancy.

Multivariable logistic regression was used to assess whether additional clinical variables beyond those provided by the MGS could improve the yield of diagnostic genotypes. Model calibration was assessed with the Hosmer-Lemeshow goodness of fit test and model discrimination was assessed by the C-statistic. Data were analyzed using R with package dplyr for calculations of genetic testing yield at various scores to determine a clinically-appropriate score cutoff that improves genetic testing yield without missing diagnostic variants. IBM SPSS Statistics (version 19) was used for multivariable logistic regression and the figure was generated with R package ggpplot2. Ethics approval for this work was obtained from the University of Washington Institutional Review Board.

Diagnostic genetic testing yield in our cohort was 34%, similar to that seen in the MGS derivation and validation cohorts (37% and 26%, respectively).^3^ The top 5 genes with diagnostic variants in our cohort included *TTN* (n = 17), *DSP* (n = 6), *LMNA* (n = 4), *RBM20* (n = 3), and *FLNC* (n = 3).

In our U.S. cohort, a MGS value of ≥1 captured all patients with diagnostic tests (Figure 1) as no individual with a score of 0 (n = 11) had a causative variant. However, applying the MGS to our U.S. cohort resulted in a modest C-statistic of 0.65 (95% CI: 0.56 to 0.74). We attempted to improve the performance of the MGS by adding clinical variables known to be associated with DCM but that were not studied in Escobar-Lopez et al. Using a multivariable analysis, we calculated ORs for the following clinical variables’ association with a positive genotype: an expanded family history of nonischemic cardiomyopathy (OR: 1.58; 95% CI: 0.79, 4.16), sustained ventricular tachycardia (OR: 0.89; 95% CI: 0.25, 3.13), complete heart block (OR: 1.75; 95% CI: 0.31, 9.96), and peripartum cardiomyopathy (1.33; 95% CI: 0.34, 5.19). However, adding these variables to the MGS did not significantly improve model performance (C-statistic = 0.68; 95% CI: 0.59-0.77). The multivariable model had reasonable goodness of fit as indicated by the statistically nonsignificant *P* value for the Hosmer-Lemeshow test (*P* = 0.38).

**Figure 1:**
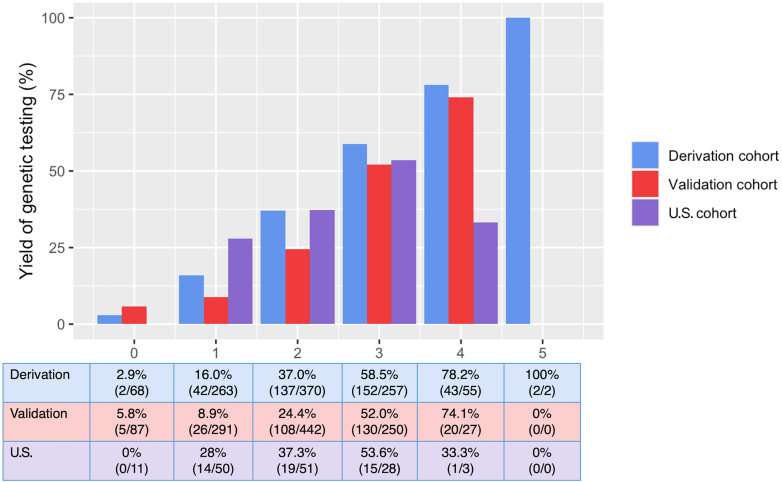
**Causative Genotype Yield by Madrid Genotype Score in Different Cohorts** Percentage of dilated cardiomyopathy patients with a causative genotype for each Madrid Genotype Score value across cohorts. The U.S.-based cohort described in this study is compared to the European-based derivation and validation cohorts described in Escobar-Lopez et al. Comparisons across score values illustrate the relationship between increasing Madrid Genotype Score and diagnostic yield in independent cohorts.

Notably, our study is limited by referral bias as the patients in the cohort had all been seen in genetics clinic. Other limitations include single-center study, incomplete capture of negative genetic test reports in the BBI-CVD, and a small sample size of 143. Despite these limitations, we externally validated the MGS in a U.S. DCM cohort. We found that applying the MGS to our U.S. cohort resulted in a C-statistic of 0.65 (95% CI: 0.56-0.74), which is not statistically different to the C-statistic of 0.74 reported in the Escobar-Lopez et al. study. Yield of diagnostic testing was similar between the cohorts up to a score value of 3 (Figure 1). MGS value > 3 had few patients in our cohort (n = 3) making comparisons less meaningful. Although we attempted to improve model performance by including additional clinical variables associated with DCM, we were not able to significantly improve diagnostic yield in our cohort. This may be due to the low prevalence of some of the conditions in our cohort, such as peripartum cardiomyopathy, which only had 10 individuals, so additional study may be warranted, especially for this variable given its known genetic association.^5^ A possible threshold for genetic testing referral could be a MGS value of ≥1 as this captures all individuals with diagnostic tests in the U.S. cohort; however, this is limited by the low numbers of individuals with a MGS value of 0 (n = 11), potentially due to referral bias. Further studies are needed to clarify the false-negative rate when using a threshold of ≥1 to refer patients for genetic testing.

In summary, in a U.S. single-center DCM cohort, the MGS achieved modest diagnostic genetic testing yields that were comparable to those reported in European cohorts. Although a score ≥1 identified all diagnostic genotypes in the U.S. cohort, additional validation is needed to support broader clinical implementation of this threshold.

## Funding support and author disclosures

Dr Yang is supported by 10.13039/100000002NIH R01HL171174, Veteran’s Administration I01BX006428, and IK2BX004642. Dr Stergachis was supported by 10.13039/100000002NIH grant R01HG013025 and a Brotman Baty Institute for Precision Medicine Catalytic collaboration grant; he holds a Career Award for Medical Scientists from the Burroughs Wellcome Fund; and he is a Pew Biomedical Scholar. Dr Resnick: This work was supported by the University of Washington Internal Medicine Residency. Dr Nazer was supported by R01HL172820. All other authors have reported that they have no relationships relevant to the contents of this paper to disclose.
